# Role of mothers’ fear of negative evaluation and perceived parenting competence on early child development: a longitudinal examination from ante- to postnatal period

**DOI:** 10.1186/s12884-026-08989-3

**Published:** 2026-03-28

**Authors:** Satoko Sasagawa, Chika Yokoyama, Haruna Irino, Aiko Okatsu, Yasue Mitamura, Chika Kubota, Ayako Kanie, Sayaka Aoyama, Miyuki Makino, Aiichiro Nakajima, Yaeko Kataoka, Masaru Horikoshi, Hironori Kuga, Masaya Ito

**Affiliations:** 1 Faculty of Psychology, Mejiro University, Tokyo, Japan; 2 National Center for Cognitive Behavior Therapy and Research, National Center of Neurology and Psychiatry, Tokyo, Japan; 3 School of Nursing, Japanese Red Cross Toyota College of Nursing, Tokyo, Japan; 4 Brain Bioregulatory Science, Cooperative Graduate School, The Jikei University Graduate School of Medicine, Tokyo, Japan; 5 Department of Women’s Psychiatry, Integrated Center for Women’s Health, National Center for Child Health and Development, Tokyo, Japan; 6 National Institute of Mental Health, National Center of Neurology and Psychiatry, Tokyo, Japan; 7 Department of Child Psychiatry, Tokyo University, Tokyo, Japan; 8 Graduate School of Nursing Science, St. Luke’s International University, Tokyo, Japan; 9 Nakama Clinic, Okinawa, Japan; 10 Faculty of Human Sciences, Musashino University, Tokyo, Japan

**Keywords:** Fear of Negative Evaluation (FNE), Perceived parenting competence, Perinatal period, Child development, Random Intercept Cross-Lagged Panel Modeling (RI-CLPM)

## Abstract

**Background:**

Fear of negative evaluation (FNE) has been linked to dysfunctional parenting in childhood, but its influence during the perinatal period remains unexplored. The present study investigated whether FNE is longitudinally associated with lower perceived parenting competence and poorer child developmental outcomes as reported by mothers. It was further hypothesized that perceived criticism of one’s parenting would be related to higher levels of FNE and reduced parenting competence.

**Methods:**

A total of 1,739 pregnant women were recruited through a large internet research panel in Japan. Participants completed online surveys at four time points: during pregnancy and at 4, 26, and 56 weeks postpartum. Complete longitudinal data were available for 768 women. Measures included the Brief Fear of Negative Evaluation Scale (BFNE), the Parenting Sense of Competence Scale (PSOC), and single-item maternal reports of child developmental indicators across regulatory, motor, social, and physical health domains, and perceived criticism.

**Results:**

Random intercept cross-lagged panel modeling (RI-CLPM) showed a negative trait-level association between BFNE and PSOC (*r* = –.49, *p* < .001). Higher prenatal FNE predicted an increased likelihood that the child would require follow-up or specialist referral at the one-month health check. The associations between PSOC and child developmental ratings became strongest by 56 weeks postpartum, as did the relationships between perceived criticism and FNE.

**Conclusion:**

These findings suggest that reducing FNE during the early perinatal period may strengthen parenting competence and improve maternal perceptions of child development later in the postpartum period.

**Trial registration:**

This study was preregistered in the University Hospital Medical Information Network Clinical Trials Registry (UMIN-CTR; ID: UMIN000044079, Date of registration: April 30, 2021).

**Supplementary Information:**

The online version contains supplementary material available at 10.1186/s12884-026-08989-3.

## Highlights

• Fear of negative evaluation was stably linked to lower perceived parenting competence

• High fear of negative evaluation and low parenting competence was associated with poorer maternal ratings of child developmental indicators

• Perceived criticism was associated with high fear of negative evaluation and low perceived parenting competence

• The associations among variables were strongest at 56 weeks postpartum

**Supplementary Information:**

The online version contains supplementary material available at 10.1186/s12884-026-08989-3.

## Introduction

The perinatal period represents a critical life transition in which women assume the new role of caring for their infants [34, 48]. From the time they discover their pregnancy, women become responsible not only for themselves but for the well-being of the fetus as well. Daily life becomes constrained by various requirements, such as adhering to dietary recommendations and attending regular medical checkups. After childbirth, mothers must continue to protect and nurture their infants while maintaining preexisting family and occupational roles. Adapting to the complex and multifaceted demands of pregnancy, childbirth, and early parenting is therefore an immense psychological and practical challenge.

The challenges associated with adapting to pregnancy, childbirth, and early parenting may vary across sociocultural contexts. In Japan, a high-income country with one of the world’s largest economies and very low infant mortality rates [50], 61.1% of parents nevertheless report that having and raising children is difficult, compared with 2.1% in Sweden, 17.6% in France, and 22.8% in Germany [5]. Contributing factors include the concentration of childcare responsibilities on mothers, limited paternal involvement associated with long working hours among fathers, and strong social expectations that mothers anticipate and manage children’s needs [16]. As a result, mothers in Japan often assume a high level of responsibility for childcare, which may place particular demands on their parenting experience.

Perceived parenting competence is a cognitive-emotional construct referring to parents’ evaluations of their abilities as caregivers. It encompasses beliefs about one’s capacity to positively influence a child’s development as well as satisfaction with the parenting role [8, 39]. A systematic review by Albanese et al. [2] shows that perceived parenting competence is a significant predictor of parental mental health, parenting behaviors, and children's mental and physical health outcomes. Specifically, perceived competence has been associated with more effective and responsive parenting [19], greater parental involvement [42], lower risk of postpartum depression and anxiety [6, 13, 26], more frequent engagement in child health-promoting behaviors [22, 23], and reduced risk of developmental delays [7]. Given its broad implications for maternal well-being and child development, enhancing parenting competence is an important goal for promoting perinatal mental health.

The Parenting Sense of Competence scale (PSOC) is among the most widely used instruments for assessing perceived parenting competence in both research and clinical settings [25]. Originally developed by Gibaud-Wallston and Wandersman [17] and subsequently revised by Johnston and Mash [24], the PSOC is a self-report measure designed to assess parents’ perceived competence and efficacy in child rearing. In their evaluation of the scale’s factor structure using a sample of parents with preschool- and school-aged children (4–9 years), Johnston and Mash [24] identified a two-factor model comprising Satisfaction and Efficacy. Since then, the PSOC has been translated into multiple languages including Japanese [1] and has undergone extensive validation. While several studies have supported the original two-factor structure (e.g., [39, 40]), others have identified a three-factor solution consisting of Satisfaction, Efficacy, and Interest [10, 18, 44]. The PSOC has been applied across diverse child age groups, including mothers during the perinatal period. It has demonstrated associations with depressive symptoms, self-esteem, and social support (e.g., [27]) and has also been used to evaluate the effectiveness of parent training interventions [4, 15].

Fear of negative evaluation (FNE), the persistent concern about being judged unfavorably by others [49], has also played an important role in parenting. For example, FNE was associated with heightened preoccupation about one’s child being criticized and with intrusive or negative parenting behaviors (e.g., [9]). Such behaviors are known risk factors for a wide array of child internalizing disorders (e.g., [20]). In addition, FNE has been linked to elevated maternal guilt and shame [30] and may function as a barrier to accessing support during the postpartum period [11]. Thus, FNE may not only contribute to maternal psychological distress but to adverse child physical and psychological outcomes, potentially through its influence on parenting cognitions, behaviors, and reduced support utilization.

Perceived criticism may have an impact on both FNE and perception of parenting competence. External evaluations and comments about one’s parenting have been shown to shape parents’ self-perceptions of competence [8]. Verhage et al. [47] found that first-time mothers with lower resilience to negative feedback reported lower parenting competence when caring for infants with difficult temperaments, whereas those with higher resilience were less affected. Taken together, these findings suggest that mothers with high FNE are particularly vulnerable to the effects of criticism, placing them at greater risk of diminished perceived parenting competence.

Despite this accumulating evidence, few studies have examined the longitudinal interplay between parenting competence, FNE, and child development. Moreover, the potential role of perceived criticism has rarely been explored in longitudinal frameworks. The present study, therefore, aims to clarify the dynamic associations among FNE, perceived parenting competence, perceived criticism, and maternal ratings of developmental indicators across multiple postpartum time points. Specifically, we hypothesized that (a) higher FNE at a preceding timepoint would be associated with lower parenting competence at the subsequent timepoint (e.g., high FNE at one month postpartum would be associated with lower parenting competence at six months postpartum), (b) higher FNE and lower parenting competence at a preceding timepoint would predict poorer maternal ratings of child developmental indicators, and (c) perceived criticism from family members and friends or acquaintances at a preceding timepoint would be associated with higher FNE and lower parenting competence at the subsequent timepoint.

In the present study, we selected assessments points of during pregnancy and at 4, 26, and 52 weeks postpartum, representing key developmental stages in the perinatal period. The 4-week assessment captures early postpartum adjustment during the puerperal phase, characterized by biological recovery and acute mood changes. The 26-week assessment reflects consolidation of maternal role adaptation and distinguishes transient distress from persistent difficulties. The 52-week assessment represents longer-term stabilization of maternal mental health and caregiving patterns and corresponds to the conventional upper boundary of the postpartum period. Together, these time points allow examination of acute, adaptive, and longer-term trajectories of maternal and child functioning.

## Methods

### Study design and participants

This study was conducted as part of a research project entitled “The Longitudinal Observational Study for Mental Health in Women in the Perinatal Periods.” The research was carried out in accordance with the Declaration of Helsinki and the Ethical Guidelines for Medical and Biological Research Involving Human Subjects issued by the Ministry of Health, Labour and Welfare of Japan. Ethical approval was obtained from the institutional review board of the National Center of Neurology and Psychiatry (initial approval: January 14, 2021; approval number: A2020-119; final approval: May 12, 2023; approval number: B2023-024).

The study was registered with the University Hospital Medical Information Network Clinical Trials Registry (UMIN-CTR), which complies with International Committee of Medical Journal Editors (ICMJE) and World Health Organization International Clinical Trials Registry Platform (WHO ICTRP) standards (UMIN ID: UMIN000044079; registration date: April 30, 2021).

A total of 1,739 pregnant women were recruited from panelists registered with Rakuten Insight Inc., a major Japanese internet marketing research firm. Eligible participants were pregnant women who provided informed consent, were fluent in Japanese, and were able to read and understand the study information presented on the online explanation screen. Several procedures were implemented to enhance response authenticity and reliability. First, the survey was administered through a registered panel in which each participant held a unique account identifier managed by the survey company. The company maintained demographic information (e.g., gender, occupation, education, socioeconomic status, and family composition), updated periodically, and a dedicated panel of pregnant women was used for this study. This enabled recruitment of participants who were pregnant at enrollment and allowed survey distribution at each of the four planned assessment waves at the intended timing. Second, each survey was accessible only during a designated response window (e.g., four weeks postpartum for T2), after which the form was closed and could no longer be accessed; participants who did not respond within the time frame were considered dropouts. Participation at each wave required logging in through the same registered account and responding within the specified data-collection period, ensuring that assessments were completed by the same individuals at the intended time points. Third, data-screening procedures were applied to identify potentially unreliable responses, including exclusion of cases with implausibly short completion times or inconsistent or patterned responding, which are commonly used indicators of inattentive responding in online research.

Participants completed online surveys at four time points: during pregnancy (T1), and at 4 (T2), 26 (T3), and 56 weeks postpartum (T4). A subsample of 768 women provided complete data across all time points. Data collection was carried out in two waves between July 2021 and November 2023. 

### Measures

The measures used in the present study were as follows:

1. Brief Fear of Negative Evaluation Scale (BFNE) [29, 46]: To measure fear of negative evaluation, BFNE, as standardized for the Japanese population, was measured at T1-T4. The scale consisted of 12 items rated from 1 = Not at all characteristic of me to 5 = Extremely characteristic of me. The excellent reliability and validity of the Japanese BFNE have been reported [46], and the scale is widely used across various Japanese populations. Cronbach’s alpha for the present sample was 0.90 at T1, 0.89 at T2 and T3, and 0.88 at T4.
2. The Parenting Sense of Competence Scale (PSOC) [17, 24]: PSOC was used to measure perceived parenting competence and efficacy. The Japanese version was developed by Abe-Kawamoto and Kobayashi [1] in samples of perinatal mothers, consisting of 14 items loading onto two subscales: efficacy and satisfaction. Each item is evaluated on a 6-point scale ranging from 1 = strongly disagree to 6 = strongly agree. Higher scores indicate greater perceived competence. The original Japanese validation study reported acceptable reliability and validity, with a Cronbach's alpha of 0.74 [1]. In the present study, PSOC was measured at T2-T4, and its Cronbach’s alpha was 0.68 at T2, 0.67 at T3, and 0.71 at T4 for the whole scale. Although some prior studies have examined the two PSOC subscales separately (e.g., [45]), we used the total score in the current analyses for three reasons. First, using the total score enhanced stability of the RI-CLPM by reducing the number of parameters to be estimated. Second, it minimized the risk of inflated Type I error by limiting multiple testing in logistic regression and t-test analyses. Third, the approach facilitated comparison with prior longitudinal studies employing similar analytic strategies (e.g., [52]).
3. Maternal ratings of developmental indicators: Participants responded to a series of single yes/no items rating their child’s development at T2–T4. The items were organized into four domains: regulatory development, motor development, social development, and physical health. The items were derived from the Infant and Toddler Health Examination Manual [38]. In Japan, comparable items are routinely included in the Maternal and Child Health (MCH) Handbook, established under the Maternal and Child Health Act, in which caregivers report observable child behaviors and daily habits to promote developmental awareness and facilitate early screening for atypical patterns [36, 37]. Accordingly, these indicators should be interpreted as screening-level measures rather than formal assessments. Example items include “Was your child recommended for additional evaluation or follow-up at the one-month checkup?” (T2), “Does your child sit without support?” (T3), and “Does your child use simple gestures such as waving or greeting?” (T4). The full list of items used is provided in Appendix A. A total of 3 items were administered at T2, 9 items at T3, and 14 items at T4.
4. Perceived criticism from family members and friends: Participants responded to two single yes/no items assessing whether they had experienced criticism from (a) family members and (b) friends or acquaintances concerning their parenting. These items were measured at T2-T4, and the exact wording is presented in Appendix B. Consistent with prior research on perceived criticism [31, 35], we employed single-item measures; however, responses were restricted to a dichotomous (yes/no) format rather than a 1–10 Likert scale. This approach was adopted because our interest focused on the subjective presence of perceived criticism rather than its degree or impact. Given the large-scale cohort design and the extensive range of variables assessed, we aimed to include key constructs comprehensively while minimizing respondent burden and cognitive load by limiting the number and complexity of rating scales.

### Statistical analysis

To evaluate potential attrition bias, baseline characteristics were compared between participants who completed all follow-up assessments and those who discontinued at some point after the first survey. Consistent with prior analyses using the same cohort [28], differences between completers and dropouts in demographic and psychological variables at baseline were generally small, suggesting limited evidence of substantial systematic selection effects. Specifically, no significant differences were found in FNE scores at T1 (*t*(1737) = 0.83, *p* = 0.41) or PSOC scores at T2 (*t*(1373) = 0.37, *p* = 0.71). The only demographic differences identified were that completers were younger (mean difference = − 0.75 years, *p* < 0.001), worked fewer hours per day (mean difference = − 0.42 h,* p* = 0.018), and had partners with more years of education. Because each survey wave required complete responses, there were no item-level missing data within waves; thus, analyses were conducted using all available cases at each time point.

Random Intercept Cross-Lagged Panel Modeling (RI-CLPM; [21]) was used to examine the longitudinal association between the T2 to T4 scores of FNE and PSOC. We employed the RI-CLPM because it allows the examination of stable, individual differences that are consistent across time (“trait”) and within-person fluctuations or deviations of a variable from an individual's average (“state”). A series of logistic regression analyses examined whether FNE and PSOC at a given time point predicted child development outcomes at the subsequent time point (i.e., T1 FNE predicting T2 outcomes, and T2-T3 FNE and PSOC predicting T3-T4 outcomes). Independent samples t-tests were utilized to compare FNE and PSOC scores between participants who reported experiencing criticism from family and friends prior to the assessment and those who reported no such experiences.

To address multiple testing, *p*-values were adjusted using the Benjamini–Hochberg false discovery rate (FDR) procedure. For logistic regression analyses, outcomes were grouped into families defined by developmental domain at each assessment wave, and FDR correction was applied within each domain-by-time family. For independent-samples t-tests, the eight comparisons were treated as a single family of parallel exploratory tests, and FDR correction was applied across all eight p-values. Both unadjusted and FDR-adjusted p-values are reported. Data were analyzed using Mplus Version 8.5 (Muthén & Muthén, Los Angeles, CA) for the RI-CLPM, and IBM SPSS Statistics Version 29 (IBM Corp., Armonk, NY, USA) for the logistic regression and t-test analyses.

## Results

### Participant characteristics

At the time of recruitment, participants had a mean age of 31.95 years (*SD* = 4.32). The average number of children during pregnancy (excluding the current fetus) was 0.71 (*SD* = 0.86). For 47.4% of participants (*n* = 364), this constituted their first child. Most participants were married or with a partner (98.7%, *n* = 758). The median annual household income fell within the 5–8 million yen range (approximately 33,000–52,000 USD). In terms of educational attainment, 1.2% (*n* = 9) had completed junior high school or lower, 13.9% (*n* = 107) were high school graduates, 27.8% (*n* = 214) had completed vocational or junior college education, 54.2% (*n* = 416) held a university degree, and 2.9% (*n* = 22) had obtained a graduate degree. Given that the median annual income in Japan is approximately 4.1 million yen [33] and that the university enrollment rate was around 50% when participants were 18 years old [32], the sample appears to be characterized by relatively high income and educational attainment compared with the general Japanese population.

### Longitudinal association between FNE and PSOC

The RI-CLPM model showed excellent fit to the data (CFI = 0.997, TLI = 0.992, RMSEA = 0.04). At the trait level, measurements of FNE and PSOC were stable; the standardized estimates for FNE ranged from 0.85 to 0.86 and for PSOC from 0.69 to 0.75, all highly significant (*p* < 0.001), suggesting the validity of applying the RI-CLPM model.

Autoregressive paths indicated that after accounting for trait-level correlation, FNE scores at preceding time points did not significantly predict subsequent scores (T2 → T3, T3 → T4; all *ps* > 0.40). For PSOC, there were significant effects from T2 to T3 (*β* = 0.13, *p* < 0.001). and from T3 to T4 (*β* = 0.12, *p* < 0.01). These results indicate that while there is a within-person association between each time points for PSOC, no such association can be identified for FNE apart from the trait-level consistency.

As for cross-lagged effects, within-person fluctuations in FNE did not significantly predict subsequent parenting competence and vice versa (all *ps* > 0.25). A significant negative residual correlation was found at T4 (*r* = 0.17, *p* < 0.01), indicating that the within-person fluctuations at this time point correlated more strongly than the other time points.

In sum, at the trait level, there was a moderate association between FNE and parenting competence. Perceived “state” parenting competence was predicted by the score at the preceding time point, suggesting that parenting skills are acquired over time and remain considerably stable. It was also suggested that the relationship between FNE and parenting competence may become stronger over time, given the residual correlation at T4 between the two variables.

### The predictive values of FNE and PSOC on child development

A series of binary logistic regression analyses were conducted to test whether FNE and PSOC at the preceding time point predicted maternal ratings of child development. Both FNE and PSOC scores were standardized and, where applicable, entered simultaneously as continuous predictors. For T2 child development outcomes, only FNE at T1 was included, as PSOC was not assessed prenatally. The results are summarized in Table 1. Variance inflation factors (VIFs) for all predictors were below 2.0, indicating no concerns regarding multicollinearity.

**Table 1:** Binary logistic regression predicting developmental outcomes from FNE and PSOC

Outcome	Predictor	*B*	SE	Wald	OR	95% CI for OR	*p*	FDR-adjusted *p*	Nagelkerke’s *R*^*2*^	AUC
T2: Physical health										
Follow-up/referral to specialist at 1-mo check-up	FNE (T1)	0.29	0.12	6.19	1.34	1.06–1.69	0.013	N/A	0.01	0.59
T3: Regulatory development										
Cry out at night	FNE (T2)	0.08	0.07	1.47	1.08	.95–1.24	0.226	0.678	0.04	0.60
	PSOC (T2)	−0.30	0.07	19.07	0.74	.65-.85	0.000	0.000		
T3: Motor development										
Sit without support	FNE (T2)	−0.16	0.07	5.82	0.85	.75-.97	0.016	0.048	0.02	0.57
	PSOC (T2)	0.12	0.07	3.49	1.13	.99–1.29	0.062	0.186		
T3: Physical health										
Diarreha	FNE (T2)	−0.04	0.13	0.08	0.96	.74–1.25	0.775	0.775	0.02	0.58
	PSOC (T2)	−0.35	0.14	6.68	0.70	.54-.92	0.010	0.020		
Skin problems	FNE (T2)	0.17	0.07	6.95	1.19	1.05–1.35	0.008	0.016	0.02	0.57
	PSOC (T2)	−0.11	0.07	2.52	0.90	.79–1.03	0.113	0.226		
T4: Regulatory development										
Cry out at night	FNE (T3)	0.05	0.08	0.33	1.05	.90–1.22	0.564	0.846	0.02	0.55
	PSOC (T3)	−0.20	0.08	6.44	0.82	.70-.96	0.011	0.033		
T4: Motor development										
Pull to stand	FNE (T3)	0.01	0.21	0.00	1.01	.67–1.52	0.982	0.982	0.05	0.68
	PSOC (T3)	0.62	0.21	8.32	1.85	1.22–2.81	0.004	0.024		
Cruise along furniture	FNE (T3)	0.01	0.15	0.01	1.01	.75–1.37	0.930	1.000	0.02	0.60
	PSOC (T3)	0.36	0.16	5.45	1.44	1.06–1.95	0.020	0.060		
T4: Social development										
Use simple social gestures such as waving, greeting	FNE (T3)	0.18	0.11	2.77	1.20	.97–1.48	0.096	0.192	0.01	0.58
	PSOC (T3)	0.24	0.11	5.05	1.27	1.03–1.57	0.025	0.050		
T4: Physical health										
Skin problems (T4)	FNE (T3)	0.19	0.08	5.44	1.21	1.03–1.43	0.020	0.040	0.01	0.51
	PSOC (T3)	−0.02	0.08	0.08	0.98	.83–1.15	0.780	0.780		

*Abbreviations*: *SE* Standard error, *OR* Odds ratio, *95%CI* 95% confidence interval, *AUC* Area under the curve, *1*-*mo* one month, *T1* during pregnancy, *T2* 4 weeks postpartum, *T3* 26 weeks postpartum, *T4* 56 weeks postpartum, *FNE* Fear of Negative Evaluation, *PSOC* The Parenting Sense of Competence Scale

Although the overall model fit and discrimination were modest (Nagelkerke’s *R*^*2*^ = 0.01–0.05, AUC = 0.51–0.68), the direction of effects was consistent with theoretical expectations, suggesting that FNE and PSOC contribute to maternal ratings of child development to an extent. Given the number of related developmental outcomes tested, false discovery rate (FDR) correction was applied within developmental domains. Findings that did not remain significant after FDR correction were interpreted as preliminary.

Higher prenatal FNE predicted a greater likelihood of the child requiring follow-up or referral to a specialist at the one-month checkup, and higher postnatal FNE was associated with reports of skin problems at both T3 and T4, as well as worse ratings in motor development at T3 (sit without support). In contrast, higher PSOC predicted greater regulatory (fewer reports of night crying at T3 and T4), motor (pulling to stand and cruising at T4) and social development (use of simple gestures at T4), and better physical health ratings (less diarrhea at T3). Collectively, associations between earlier PSOC and maternal reports of child developmental indicators appeared more pronounced at one year postpartum than at six months.

### Differences in FNE and PSOC scores by perceived criticism

A series of independent sample t-tests compared subsequent FNE and PSOC levels between participants who reported prior criticism from family members or friends/acquaintances and those who did not. The results are presented in Table 2. After FDR correction, three of the 8 comparisons remained significant. Participants who reported being criticized by family members at the prior time point scored lower on PSOC at T3 and T4, and higher on FNE at T4. Criticism from friends and acquaintances showed only marginal effects on PSOC at T4, and no significant group differences emerged at T3, possibly reflecting the small number of participants endorsing such criticism (fewer than 5% of the total sample). Overall, these findings suggest that feedback from family members influences changes in FNE and PSOC over time, with effect sizes (Cohen’s d) for FNE increasing at the later time point.

**Table 2:** Group differences in FNE and PSOC scores at the preceding time point by the presence of criticism

Outcome	Group	*n*	mean	SD	*t*	*p*	FDR-adjusted *p*	Cohen’s *d*
FNE (T3)	Criticism from family (T2)							
	Y	120	36.24	10.78	−1.69	0.081	0.108	−0.17
	N	919	34.55	9.86				
	Criticism from friends (T2)							
	Y	15	34.87	12.71	−0.05	0.963	0.963	−0.01
	N	1024	34.75	9.94				
PSOC (T3)	Criticism from family (T2)							
	Y	120	50.95	7.58	3.09	0.002	0.008	0.30
	N	919	53.42	8.31				
	Criticism from friends (T2)							
	Y	15	51.13	8.28	0.95	0.345	0.394	0.25
	N	1024	53.17	7.01				
FNE (T4)	Criticism from family (T3)							
	Y	130	36.67	10.39	−2.77	0.006	0.016	−0.27
	N	638	34.08	9.58				
	Criticism from friends (T3)							
	Y	37	37.57	10.02	−1.95	0.051	0.102	−0.33
	N	731	34.36	9.73				
PSOC (T4)	Criticism from family (T3)							
	Y	130	50.82	8.79	3.25	0.001	0.008	0.31
	N	638	53.39	8.12				
	Criticism from friends (T3)							
	Y	37	50.38	7.54	1.94	0.052	0.083	0.33
	N	731	53.09	8.31				

*Abbreviations*: *n* number of participants, *SD* standard deviation, *Cohen's* *d* = Cohen’s standardized mean difference effect size, *FNE* Fear of Negative Evaluation Scale, *PSOC* The Parenting Sense of Competence Scale, *T3* 26 weeks postpartum, *T4* 56 weeks postpartum, *Y* those who responded "yes" to the question, *N* those who responded "no" to the question

## Discussion

The present study examined the longitudinal relationships between FNE, PSOC, maternal perceptions of child development, and perceived criticism regarding parenting across the perinatal period. While no temporal cross-lagged effects were observed, higher FNE was associated with lower perceived parenting competence, and the relationship between the two traits remained stable. Although FNE is typically considered a modifiable construct often targeted in cognitive-behavioral therapy for social anxiety disorder (e.g., [3]), the current findings suggest that FNE levels remains relatively stable throughout the perinatal period. In contrast, PSOC demonstrated significant within-person variability, with scores at each time point directly influencing subsequent scores. The association between FNE and PSOC was strongest at 56 weeks postpartum (T4), indicating that the link between these constructs may intensify as the child grows.

The hypotheses concerning the relationship between FNE and PSOC with maternal ratings of child development were generally supported, though the effects were relatively weak. As was predicted, higher FNE was associated with less favorable ratings, whereas higher PSOC was linked with more adaptive mother reports. Although model fit indices and discrimination were modest, the overall direction and consistency of effects align with the theoretical framework suggesting that evaluative fears and perceived childrearing efficacy are related to mothers’ perceptions of their children’s development. Specifically, elevated prenatal FNE was associated with a greater likelihood that infants were reported as requiring follow-up or specialist referral at the one-month health checkup. Given that the present study relied on single-item maternal ratings, these findings should be interpreted cautiously. Nevertheless, they are broadly consistent with prior meta-analytic evidence indicating that maternal anxiety during pregnancy is associated with increased risk of preterm birth and low birth weight [12]. In this review, the authors suggested that heightened prenatal anxiety may be linked to elevated stress hormones, potentially increasing vulnerability to adverse birth outcomes.

Postnatal FNE was also associated with later reports of skin problems. Yonezawa et al. [51] reported that infant skin conditions are related to poorer maternal quality of life, even when symptoms are not severe. The present findings suggest that FNE may show a comparable association with mothers’ perceptions of such health concerns.

Greater PSOC was associated with better maternal ratings in all four domains, suggesting that mothers who feel more capable may engage in more responsive caregiving and assess their children’s development more positively. Notably, these associations were more evident at 56 weeks postpartum (T4) than at 26 weeks (T3), suggesting that the influence of perceived parenting competence on maternal ratings of developmental indicators becomes increasingly pronounced over time.

Group comparisons revealed that perceived criticism from family members, but not from friends, was associated with higher levels of FNE and lower PSOC. These findings suggest that family communication patterns may represent a potentially relevant target for future intervention research. Furthermore, the larger effect size observed at 56 weeks postpartum (T4) for FNE may indicate that associations between perceived criticism and evaluative concerns become more pronounced over time. Given that family members often experience heightened stress and limited personal time following childbirth, psychoeducational programs aimed at reducing critical communication and alleviating FNE could help enhance maternal competence and, ultimately, promote positive ratings of child development.

Liss et al. [30], in a cross-sectional study of mothers with children aged five years and under, demonstrated that FNE moderated the relationship between maternal guilt and shame and feelings of self-discrepancy. The present study extends this line of research by showing, through longitudinal data, that the influence of FNE emerges much earlier in the course of motherhood and may be amplified by criticism from family members. Previous research has further indicated that shame proneness predicts less favorable attitudes toward help-seeking during the postnatal period [14] and that FNE is linked to reduced help-seeking among mothers experiencing postpartum mood disorders [43]. Collectively, these findings underscore the social and clinical importance of implementing targeted interventions early in the perinatal period to mitigate the adverse effects of evaluative fears and promote maternal well-being.

Several limitations of the present study should be acknowledged. First, all data were collected through internet-based self-report questionnaires, which may be subject to reporting biases, unreliable responses, and shared method variance. Future studies could benefit from reports from partners and healthcare professionals. In particular, the inclusion of fathers’ perspectives is important, as previous studies indicate that fathers’ behaviors and attitudes influence both maternal mental health and childrearing (e. g., [41]). Accordingly, examining the family as an integrated system represents an important direction for future work. Second, child developmental outcomes and perceived criticism were assessed using single-item maternal reports. These items were derived from routine Japanese infant health examinations and previous studies employing similar research designs; however, its validity and reliability need to be interpreted with caution, and future research should replicate these associations using observational measures and validated multi-item developmental assessments. Third, although longitudinal data were used, causal conclusions cannot be drawn. Intervention or experimental studies are needed to determine whether reducing FNE enhances parenting competence and subsequently improves mother evaluation of child developmental outcomes. Fourth, attrition may have affected the findings. As detailed elsewhere [28], participants who completed all waves differed at baseline from those who discontinued participation in terms of age, working hours, and partner educational attainment. In addition, the use of an internet-based sampling strategy may have underrepresented certain populations. Together, these factors may have introduced selection bias and limited the generalizability of the results. Notably, the stronger associations observed at T4 between child outcomes and FNE/PSOC may partly reflect the impact of attrition bias. Despite these limitations, the findings highlight the importance of addressing maternal social-cognitive vulnerability in early parenting support, offering promising directions for future prevention and intervention efforts.

## Conclusion

The present findings indicate that FNE and PSOC are interconnected psychological constructs associated with early maternal ratings of child development. Interventions aimed at reducing maternal sensitivity to negative evaluation and enhancing parenting competence, particularly within family contexts, may help promote healthier adjustment and perceptions of child development during the perinatal period. Although effect sizes were small, the results contribute to understanding how social-cognitive factors and criticism shape maternal confidence and early developmental indicators.

## Supplementary Information

Supplementary Material 1.

## Data Availability

The datasets generated and/or analyzed during the current study are not publicly available due to ethical restrictions but are available from the corresponding author on reasonable request.
