# Supplementary material for: Role of mothers’ fear of negative evaluation and perceived parenting competence on early child development: a longitudinal examination from ante- to postnatal period
Source: BMC Pregnancy Childbirth. 2026 Mar 28;26:498. doi: 10.1186/s12884-026-08989-3 (PMC13151394; doi:10.1186/s12884-026-08989-3)
Supplement: Supplementary file 1 — Supplementary Material 1. [file 12884_2026_8989_MOESM1_ESM.docx]

Appendix A

Single-item questions regarding child development

【T2】

Regulatory development

1. Do you breastfeed at a regular interval?

2. Does your child wake up crying at night?

Physical health

3. Was your child recommended for additional evaluation or follow-up at the one-month checkup?

【T3】

Regulatory development

1. Do you breastfeed at a regular interval?

2. Does your child wake up crying at night?

3. Has your child started weaning (solid foods)?”

Motor development

4. Has your child achieved head control?

5. Does your child roll over?

6. Can your child sit without support?

Social development

7. Does your child smile a lot when you interact with them?

Physical health

8. Does your child often have diarrhea?

9. Does your child have skin problems?

【T4】

Regulatory development

1. Do you breastfeed at a regular interval?

2. Does your child wake up crying at night?

3. Has your child started weaning (solid foods)?”

Motor development

4. Does your child roll over?

5. Does your child sit without support?

6. Does your child crawl?

7. Does your child pull to stand?

8. Does your child cruise along the furniture?

9. Does your child walk well on his/her own?

Social development

10. Does your child use simple gestures such as waving or greeting?

11. Does your child say a few meaningful words such as ‘bye-bye’ or ‘hello’?”

Physical health

12. Does your child often have diarrhea?

13. Does your child have skin problems?

Appendix B

Items measuring perceived criticism from family members and friends

1. I was criticized by my family about my parenting.

2. I was criticized by friends or acquaintances about my parenting.
